# IT-SVO: Improved Semi-Direct Monocular Visual Odometry Combined with JS Divergence in Restricted Mobile Devices

**DOI:** 10.3390/s21062025

**Published:** 2021-03-12

**Authors:** Chang Liu, Jin Zhao, Nianyi Sun, Qingrong Yang, Leilei Wang

**Affiliations:** 1 School of Mechanical Engineering, Guizhou University, Guiyang 550025, China; gs.changliu20@gzu.edu.cn (C.L.); gs.nysun19@gzu.edu.cn (N.S.); gs.qryang18@gzu.edu.cn (Q.Y.); gs.wangll19@gzu.edu.cn (L.W.); 2 Key Laboratory of Advanced Manufacturing Technology, Ministry of Education, Guizhou University, Guiyang 550025, China

**Keywords:** SLAM, localization, information theory, JS divergence, tracking

## Abstract

Simultaneous localization and mapping (SLAM) has a wide range for applications in mobile robotics. Lightweight and inexpensive vision sensors have been widely used for localization in GPS-denied or weak GPS environments. Mobile robots not only estimate their pose, but also correct their position according to the environment, so a proper mathematical model is required to obtain the state of robots in their circumstances. Usually, filter-based SLAM/VO regards the model as a Gaussian distribution in the mapping thread, which deals with the complicated relationship between mean and covariance. The covariance in SLAM or VO represents the uncertainty of map points. Therefore, the methods, such as probability theory and information theory play a significant role in estimating the uncertainty. In this paper, we combine information theory with classical visual odometry (SVO) and take Jensen-Shannon divergence (JS divergence) instead of Kullback-Leibler divergence (*KL* divergence) to estimate the uncertainty of depth. A more suitable methodology for SVO is that explores to improve the accuracy and robustness of mobile devices in unknown environments. Meanwhile, this paper aims to efficiently utilize small portability for location and provide a priori knowledge of the latter application scenario. Therefore, combined with SVO, JS divergence is implemented, which has been realized. It not only has the property of accurate distinction of outliers, but also converges the inliers quickly. Simultaneously, the results show, under the same computational simulation, that SVO combined with JS divergence can more accurately locate its state in the environment than the combination with *KL* divergence.

## 1. Introduction

As computer technology rapidly grows, multimedia applications have penetrated into almost every aspect of our daily life. In recent years, simultaneous localization and mapping (SLAM) has been a major research topic in the robotics and computer vision communities. Workspace maps and robot location information are essential to the unmanned micro-aerial vehicle (MAV) and any other robots performing tasks in unknown environments. For example, in unfamiliar indoor environments, a MAV demands real-time poses and orientation information for obstacle avoidance, motion planning, and navigation [1]. In the GPS-denied or weak GPS environment [2,3], robust and accurate state estimation is necessary to realize environmental perception and motion planning. Because of energy restriction, computing resources, and platform size, it is crucial to utilize resources to perform tasks and estimate positions reasonably [4]. Therefore, it has become an important research hot spot for localization to utilize the information from a single sensor effectively and to provide optimal self-state estimation in factories, mines, etc.

SLAM was the first approach to be adopted by mobile robotics [5,6] and is estimated by the self-state or reconstructs the unknown environment [7]. Currently, SLAM technology is gradually moving closer to lightweight applications and miniaturization. Many teams are committed to effectively integrating the SLAM method into small devices, such as embedded devices, then considering it as an underlying work [8]. However, SLAM only provides a self-state estimation for the upper applications. Our goal with SLAM is to achieve the functionality of robotics, AR/VR devices, and UAVs/UGVs, which functions involve motion, navigation [9,10], teaching, entertainment, etc. SLAM is not expected to consume all computational resources, but there is a strong demand for the miniaturization and lightweight of SLAM. Among the different sensor modalities, cameras offer the advantage of low cost but rich information. Therefore, visual SLAM solutions, in which the primary sensor is a camera, are of significant interest. Monocular cameras are one of the most common sensors found in many SLAM applications [11]. SVO [12] and ORB-SLAM [13] are typical representatives of monocular applications. The semi-direct visual odometry (SVO) algorithm uses features. However, feature-correspondence is the implicit result of direct motion estimation rather than explicit feature matching [12]. Compared with other systems, the remarkable advantages of SVO are that it allows increased speed without extracting features from each frame and provides accuracy through sub-pixel feature correspondence. Meanwhile, SVO is integrated with a probabilistic mapping method, which is robust to outlier measurements, so it can achieve real-time performance on a low-cost computing platform, like MAVs. However, the target platform for application is the top-view camera on a MAV. The object in the field of view is mainly the ground, and the camera mainly moves horizontally, up and down, which makes it perform poorly in rotation localization and easily blur. ORB-SLAM is a feature-based method and real-time graph optimization system, which has tracking, loop detection, and re-localization modules. However, the entire system uses ORB feature points for mapping and tracking, and as a result, it will consume more computing resources than using optical flow [14]. It is challenging to implement the algorithm on a microprocessor and only to calculate in real-time on the current computer architecture, while all three threads are simultaneously starting in the running process of a system. On the contrary, other solutions that provide lightweight localization, such as PTAM [15], DSO [16], VINS [17], etc., can be implemented on low-cost processors or allow the CPU to handle other tasks.

At present, there are mainly feature-based and pixel-based methods for V-SLAM. Although feature-based methods have dominated the field for a long time, in recent years, many direct, dense, and semi-dense methods have still remained popular. In feature-based visual SLAM, the map information includes localization and sparse features [18], both of which are used for better localization and mapping. Unfortunately, they are not suitable for other tasks. Nowadays, the primary feature-based method is ORB-SLAM2 [19]. Direct methods do not extract features but rather use the pixel intensities in the images directly and estimate the motion by minimizing photometric errors. LSD-SLAM [20] can build large scope maps from pixels with high gradients. However, the map estimation reduces pose-graph and achieves lower accuracy than ORB-SLAM. The current mainstream pixel-based approach is the direct sparse odometry (DSO) created by Engel et al. [16], which can compute accurate camera poses in cases of poor performances of some point detectors, and enhance the robustness in low texture regions. To make the map more detailed, Lzadi et al., constructed a dense reconstruction system with Kinect-Fusion [21]. Although the method used 3D reconstruction of small-scale scenes and individual objects, and applied the intercepted symbol distance fusion (TSDF) map results to further elevate the precision, the memory size was limited. Range and accuracy of reconstruction cannot be obtained at the same time. The hybrid system SVO [22] extracts FAST features and makes use of a direct method to track features and any pixel with non-zero intensity gradient, which optimizes camera pose through reprojection errors. SVO is particularly efficient, but as a pure VO it can only perform short-term tracking, limiting its accuracy. Therefore, this method can only be efficiently implemented on restricted mobile devices.

**Figure 1:**
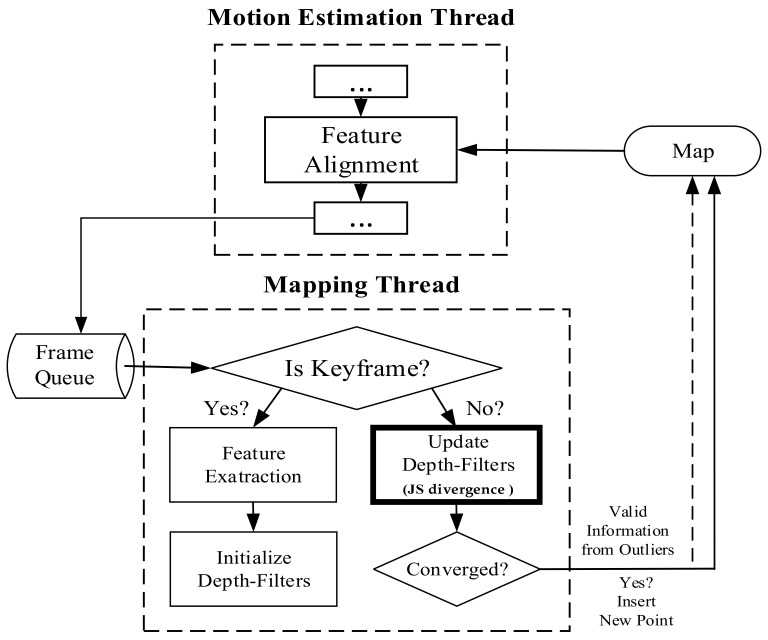
Tracking and mapping pipeline.

Simultaneously, the rapid development of science and technology has been a hot spot for multidisciplinary intersections. Information theory, probability theory, and game theory are being increasingly exploited in deep learning, state estimation, localization [23], and navigation. Reasonable inference allows machines and equipment to make optimal judgments in the face of emergencies, which increases the robustness and anti-interference of the system. In visual SLAM, LSD-SLAM combines a Kalman filter with a first-order Markov model for the location of map points in three-dimensional space. ClusterVO [24] proposed a multilevel probabilistic data association strategy for dynamic road signs. It also used Shannon entropy to express the uncertainty of map points in three-dimensional space. The clustering method is combined with road features to realize the uncertainty convergence and realize association of low-level features. Since the experiments are only for a single object and the versatility is insufficient, it has to verify the data association method under multiple categories. In the scene structure analysis of structure-SLAM [25], the author uses cross-entropy to predict the normal of the plane, which reduces the rate of mismatching to a certain extent. However, it consumes a lot of resources when removing outliers, which leads to poor real-time performance. Kullback-Leibler divergence (*KL* divergence) [26] was used to parameterize the probability in the mapping thread in SVO and achieve a balance between the inliers and outliers. Because *KL* divergence is bounded, significant differences in linearization and asymmetry of *KL* divergence can lead to gradients that cannot be updated. We are also exploring a method that is more suitable for special mobile devices. Considering the limitations of devices and environment, it is efficient and accurate in localization, so we need a more robust algorithm. Therefore, during the exploration, we finally chose Jensen-Shannon divergence (JS divergence), a deformation of *KL* divergence, with SVO instead. Figure 1 illustrates a simplified pipeline of the improved SVO, where the algorithm remains with SVO on a parallel thread, mainly for sparse mapping and localization on small devices. The tracking thread uses a semi-direct method of relative pose estimation, and the mapping thread uses the depth filter. As shown in Figure 1, the application of the depth filter in the mapping thread, and using JS divergence from information theory instead of the *KL* divergence gives better results in dealing with outliers, which is demonstrated in Section 3.

This paper takes MAV and camera as the test platform and SVO as the improved algorithm. Meanwhile, we named it IT-SVO, which stands for semi-direct visual odometry combined with information theory. We discuss the difference between *KL* divergence and JS divergence, then use JS divergence to estimate the depth and carry out the following experiments:

- The front-end tracking of PL-SVO [27,28,29] was adopted (the mapping phase is the same as SVO), which improved the robustness of the system;
- The back-end depth filter replaced *KL* divergence with JS divergence and quantified the uncertainty of tracking. The experiments showed that JS divergence improved the accuracy of system localization.
- We had performed a numerical evaluation on standard SLAM datasets (such as TUM RGB-D, EuRoC), and the results showed that the proposed method was better than the original SVO.

The content of this paper as divided as follows: We introduce the application of information theory in Section 2, compare JS Divergence and *KL* Divergence in Section 3, experimentally confirm our findings in Section 4 and summarize the work in Section 5.

## 2. Information Theory in SVO

### 2.1. Introduction of Information Theory

SVO employs a depth filter in the mapping thread, but it is not described in detail [12]. The authors of SVO describe each step of the depth filter [30]. This paper describes a three-dimensional reconstruction using the pose provided by SVO, which belongs to progressive three-dimensional reconstruction. The process is the same as the SVO depth filter, except that GPU acceleration is implemented [30].

The depth filter in SVO uses a series of front and back frames to obtain the pixel depth of the mentioned frames, but there will be errora in calculating the depth of pixels and fusing multiple depth measurements by using a series of frames for each pixel depth and a single match recovery depth. SVO considers the depth error model of pixels as a probability distribution. It has two properties: one is the depth values follow a Gauss distribution, and the other is the probability of outliers obeys a beta distribution.

### 2.2. Reasons for Choosing KL Divergence

Kullback-Leibler divergence (*KL* divergence), also known as relative entropy, was initially used to indicate the degree of loss in information transmissiona. It is used in probability theory to show the difference between two similar distributions. *KL* divergence itself is non-negative and asymmetric: $KL\left(P||Q\right)\neq KL(Q||P)$, expressed as:

(1) $$KL\left(P||Q\right)=\sum P\left(x\right)log\frac{P\left(x\right)}{Q\left(x\right)}$$

*KL* divergence is equivalent to the difference in information entropy between two probability distributions. Suppose that one of the probability distributions is a proper distribution, and the other is a theoretical (fitting). In that case, *KL* divergence represents the information loss produced by fitting of the proper distribution to the theoretical distribution. The smaller between P and Q, the closer they are. Therefore, we can use Q as a probability to estimate P. SVO constructs a depth filter by taking a proper estimation of the mapping thread.

### 2.3. KL Divergence in SVO

**Figure 2:**
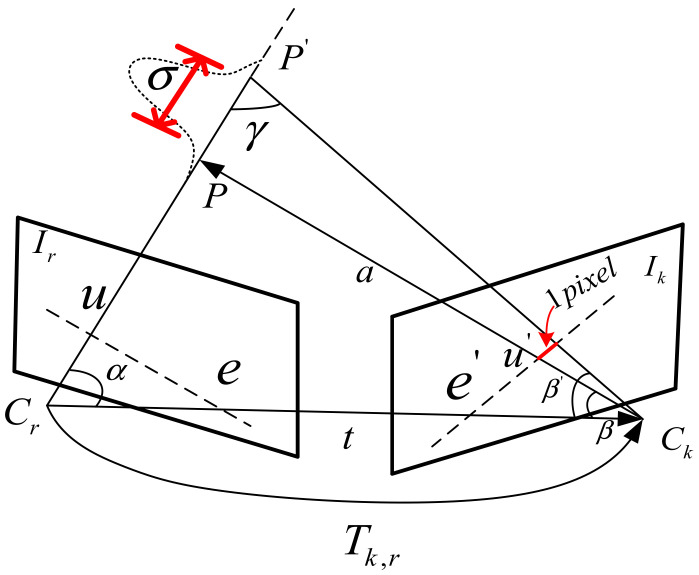
The depth uncertainty of SVO.

We know that uncertainty exists in any probability-based model. In machine learning and state estimation, the uncertainty of the model can be approximated by information theory, where the probabilities are parameterized by comparing the similarity of the measured and the estimated to obtain a more precise estimation range. The mapping phase of SVO is how to deal with the depth uncertainty caused by monocular vision as shown in Figure 2:

According to the epipolar constraint, $I_{r},I_{k}$: Two frames of images in continuous motion; $T_{k,r}$: The transformation matrix between two frames; $P,P^{\prime }$: Depth of the same point observed in different frames; σ: Because of the uncertainty of the monocular scale, here is the difference between two observation points, so multiple observations are needed to reduce the uncertainty. First, the depth filter model of SVO is:

(2) $$p\left(d_{k}|\hat{d},\rho \right)=\rho N\left(d_{k}|\hat{d},\tau_{k}^{2}\right)+\left(1-\rho \right)U\left(d_{k}|d_{min},d_{max}\right)$$

where $d_{k}$: depth measurements $\left(k=r,\ldots ,r+n\right)$; $N\left(d_{k}|\hat{d},\tau_{k}^{2}\right)$: Gaussian distribution centered on truth-value $\hat{d}$; $\tau_{k}^{2}$ is the variance; ρ: The probability of the measured value, which belongs to a good model; $d_{min},d_{max}$: The upper and lower limits of uniform distribution; Suppose $\hat{d}$, ρ is s uniform distribution. We label each depth measurement with $y_{k}$. The probability model of Equation (2) is rewritten as follows:

(3) $$p\left(d_{k}|\hat{d},\rho ,y_{k}\right)=N\left(d_{k}|\hat{d},\tau_{k}^{2}\right)U{\left(d_{k}\right)}^{1-y_{k}}$$

According to the multiplication theorems in probability theory, Equation (3) is rewritten as:

(4) $$p\left(XY,\hat{d},\rho \right)=\left[\overset{n}{\underset{k=r}{\prod }}p\left(d_{k}|\hat{d},\rho ,y_{k}\right)p\left(y_{k}|\rho \right)\right]p\left(\hat{d}\right)p\left(\rho \right)$$

Suppose there is an approximate solution $q\left(Y,\hat{d},\rho \right)$ of a posterior estimate $\;p\left(Y,\hat{d},\rho |X\right)$, which satisfies:

(5) $$q\left(Y,\hat{d},\rho \right)=q_{Y}\left(Y\right)q_{\hat{d},\rho }\left(\hat{d},\rho \right)$$

The decomposition of Equation (5) is derived from the maximum a posteriori (MAP) probability arg max[*p*(x|*v,*y)] of state estimation [31]. It is well known that in the estimation process, the maximum posterior estimation is to solve:

(6) $$\hat{x}=\text{arg}\;\text{max}\left[p\left(x|v,y\right)\right]$$

where $\;\hat{x}$: a posteriori estimate (containing the observed); x: The system state; y: The measurement; v: The input. Then arg max is obtained by rewriting the maximum a posteriori (MAP) estimate using Bayes’ rule:

(7) $$\hat{x}∶=\text{arg}\;\text{max}\left[p\left(x|v,y\right)\right]=\text{arg}\;\text{max}\left[\frac{p\left(y|v,x\right)p\left(x|v\right)}{p\left(y|v\right)}\right]=\text{arg}\;\text{max}\left[p\left(y|x\right)p\left(x|v\right)\right]$$

where we drop the denominator because it does not depend on *x*, then we get the product of two probabilities, so Equation (7) is similar to Equation (5). In SVO, depth estimation is modeled as a linear Gaussian system (LG system) because the full Bayesian posterior is precisely Gaussian [32]. Therefore, the optimization approach will find the maximum (this is mode) of Gaussian, which is the same as the mean. Finally, we will get:

(8) Hx=b

where H*:* Hessian matrix. x: Update variable. b: A vector consisting of the system state, input and measurement. Therefore, Equations (6)–(8) are expressed as converting probability parameterization to numerical calculation. When *KL* divergence of $p\left(Y,\hat{d},\rho |X\right)$ and $q\left(Y,\hat{d},\rho \right)$ is minimum value, the following conditions are satisfied:

(9) $$\ln q_{\hat{d},\rho }\left(\hat{d},\rho \right)=E_{y}\left[\ln p\left(X,Y,\hat{d},\rho \right)\right]+const$$

(10) $$\ln q_{Y}\left(\hat{d},\rho \right)=E_{\hat{d},\rho }\left[\ln p\left(X,Y,\hat{d},\rho \right)\right]+const$$

Finally, the depth model of SVO is modeled as an approximate solution of probabilistic parameterization.

## 3. JS Divergence vs. *KL* Divergence

### 3.1. Reasons for Choosing JS Divergence

Due to the non-commutative of *KL* divergence, it cannot be understood as the concept of ‘distance’. It is not the distance of two distributions in space. Still, the information loss of one distribution has to be compared with another one. Because *KL* divergence is asymmetric, for some p and q there will be exposure to $D_{KL}\left(p||q\right)\neq D_{KL}\left(q||p\right)$. This asymmetry means that choosing $D_{KL}\left(p||q\right)$ or $D_{KL}\left(q||p\right)$ can make a big difference.

During the operation of SLAM or VO, outliers are matching in data association. When iterating along with the outliers gradually, the system is likely to diverge and cause the operation to crash, so the two distributions, map points $\left(p\right)$ and noise $\left(q\right)$, are estimated. The noise also contains certain information that affects the distribution of map points. When distributing *KL* divergence and JS divergence that are far away or not overlapping, there is no mean in *KL* divergence that will be a constant, which means that the pixel is equipped with a nonzero intensity gradient. Whether machine learning or SLAM/VO, if the gradient is zero, it will produce a seven-dimensional zero space [16]. The manifestation may be abruptly reduced in the scale of the map. At the same time, it is clear from the introduction that the choices of different information theory methods will have a significant influence on the results, and that a proper information theory method will lead to a more robust and accurate distribution of the system.

### 3.2. Introduction of JS Divergence

#### 3.2.1. Application of JS Divergence in SVO

The *KL* divergence is asymmetric, and can be transformed into JS divergence with a slight modification. First, we assume that:

(11) $$JSD\left(p||q\right)=\frac{1}{2}KL\left(p||m\right)+\frac{1}{2}KL\left(q||m\right)$$

The *KL* divergence replaced by the following expression:

(12) $$JSD\left(p||q\right)=\frac{1}{2}\sum p\left(x\right)\log\left(\frac{p\left(x\right)}{\frac{p\left(x\right)+q\left(x\right)}{2}}\right)+\frac{1}{2}\sum q\left(x\right)\log\left(\frac{q\left(x\right)}{\frac{p\left(x\right)+q\left(x\right)}{2}}\right)$$

Finally:

(13) $$JSD\left(p||q\right)=\frac{1}{2}\sum p\left(x\right)\log\left(\frac{p\left(x\right)}{p\left(x\right)+q\left(x\right)}\right)+\frac{1}{2}\sum q\left(x\right)\log\left(\frac{q\left(x\right)}{p\left(x\right)+q\left(x\right)}\right)+\log2$$

That is because: $\sum p\left(x\right)=\sum q\left(x\right)=1$.

**Figure 3:**
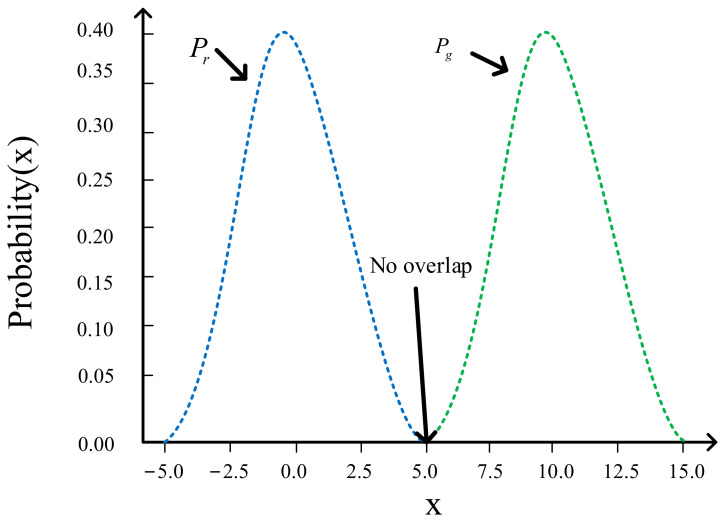
JS divergence vs. *KL* divergence.

When A and B non-overlapping, the part of left is zero as seen in Figure 3.

Let $P_{r},P_{g}$ obey the normal distribution and $\;p\left(x\right)$ be the probability when $\;P_{r}$ gets x, it will be found that there is non-overlapping between the two distributions, so on the left side of Equation (13), we knon that when $x\geq 5,p\left(x\right)\approx 0$, Equation (13) becomes:

(14) $$\frac{1}{2}\sum 0\times \log\left(\frac{0}{0+q\left(x\right)}\right)+\frac{1}{2}\sum q\left(x\right)\log\left(\frac{q\left(x\right)}{0+q\left(x\right)}\right)=0$$

When $x\leq 5,p\left(x\right)\approx 0$, Equation (11) has become:

(15) $$\frac{1}{2}\sum p\left(x\right)\log\left(\frac{p\left(x\right)}{p\left(x\right)+0}\right)+\frac{1}{2}\sum q\left(x\right)\log\left(\frac{0}{p\left(x\right)+0}\right)=0$$

Finally:

(16) $$\forall x\in R,JSD\left(p||q\right)=\log2,D_{KL}\left(p||q\right)=\emptyset$$

This is the difference between *KL* divergence and JS divergence. When the two distributions do not overlap, even centers of the two distributions are close, the *KL* divergence becomes nonsense, so the gradient is zero and cannot be updated. The JS divergence still has a numerical, which smoothed the influence of noise to a certain extent.

#### 3.2.2. Why These Two Distributions Not Overlap

**Figure 4:**
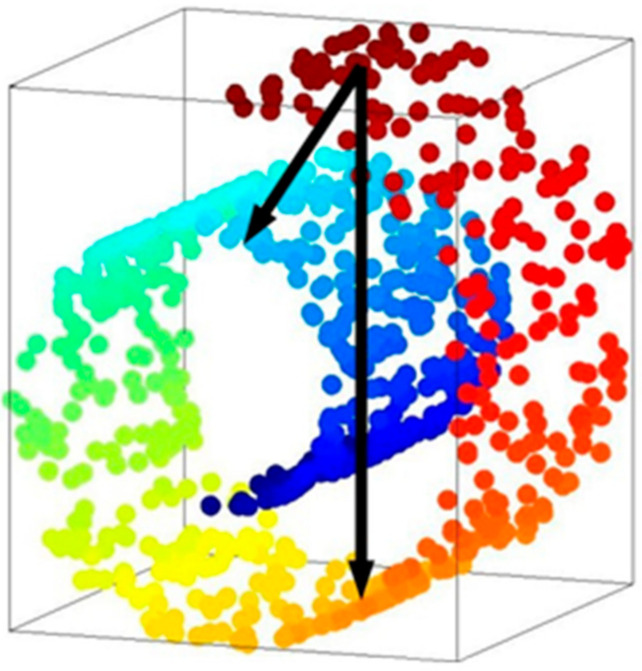
Data distribution in low-dimensional manifold (Circles of various colors in the cube represent data).

To understand the need for depth estimation is important to understand the nature of sensors. All sensors have limited precision. All measurements derived from real sensors have associated uncertainty. Therefore, there will be more noise for the camera sensor, which usefully carrying information determines the corresponding distribution of overlap. Theoretically, the real data distribution is usually a low-dimensional manifold, which means that the data does not have high-dimensional properties but exist in a low-dimensional space embedded in a high-dimensional area. In the three-dimensional space, the data is actually on a two-dimensional plane as shown in Figure 4.

In practice, the dimensional space is much more than three-dimensions, and may be hundreds of dimensions, so the data is more challenging to overlap.

### 3.3. Application of JS Divergence in SVO

We need to introduce JS divergence in the posterior estimate. Comparing Equations (9) and (10), assuming $m=\frac{1}{2}\left(p+q\right)$, and the relationship between *KL* divergence and expectation in Equation (11) we get:

(17) $$JSD\left(p||q\right)=\frac{1}{2}\left[E\left(log^{p}-log^{\frac{p+q}{2}}\right)+E\left(log^{q}-log^{\frac{p+q}{2}}\right)\right]$$

From Equation (17):

(18) $$JSD\left(p||q\right)=\log2+\frac{1}{2}\left[E\left(log^{\frac{p}{p+q}}\right)+E\left(log^{q}-log^{\frac{q}{p+q}}\right)\right]$$

The inequality of arithmetic and geometric means (Equation (19)) and the properties of the logarithmic function, lead to Equation (20):

(19) $${\left(\frac{a_{1}+a_{2}+\cdots +a_{n}}{n}\right)}^{n}\geq a_{1}+a_{2}+\cdots +a_{n}$$

(20) $$JSD\left(p||q\right)\geq w_{h}+D_{KL}\left(p||q\right)$$

**Figure 5:**
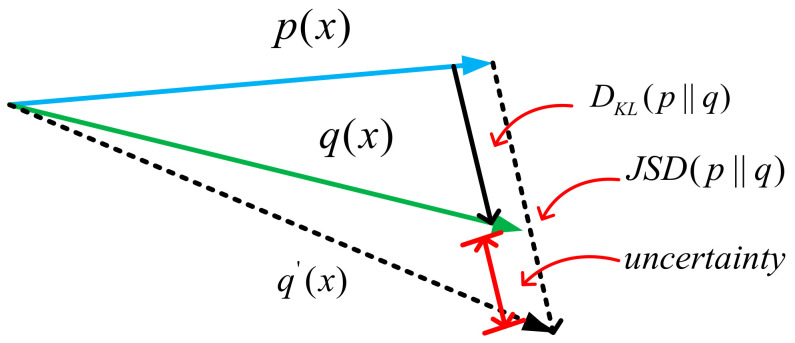
*KL* divergence and JS divergence (simplified vector diagram).

We have obtained the correspondence between JS divergence and *KL* divergence in the estimated point of SVO as seen in Figure 5.

The actual probabilities—*KL* divergence approximation probability, and JS divergence approximation probability—are denoted as $p\left(x\right),q\left(x\right),q^{\prime }\left(x\right)$, respectively, and the relationship between them is represented by vector simplification.

It is well known the measurement of probabilities in learning algorithms and SLAM/VO assumes a Gaussian distribution and has a more complex character. Finally, we need to transform the model into a continuous product of two Gaussian distributions [33]. It also contains how to transform from two different distributions into a product of two Gaussian distributions. The final model is:

(21) $$N\left(d|Z,\tau^{2}\right)N\left(Z|\mu ,\sigma^{2}\right)=N\left(d|\mu ,\sigma^{2}+\pi^{2}\right)N\left(Z|\frac{d\sigma^{2}+\mu \tau^{2}}{\tau^{2}+\sigma^{2}},\frac{\pi^{2}\sigma^{2}}{\sigma^{2}+\tau^{2}}\right)$$

Meanwhile, the covariance represents the difference between them, so the fusion strategy chooses Gaussian normalized product, and the uncertainty in the depth filter updated:

(22) $$\frac{1}{{\left(\tau^{\prime }\right)}^{2}}=\frac{1}{\tau^{2}}+\frac{1}{\tau_{h}^{2}}$$

In Equation (22), $\tau^{2}$: The variance introduced by the depth filter itself. $\tau_{h}^{2}$: The variance is introduced by the JS divergence.$\;{\left(\tau^{\prime }\right)}^{2}$: The variance after fusing the Gaussian normalized product. According to Equation (21), the second part on the right, $\tau^{2}$ in Gaussian distribution, is corresponding to Equation (22). Therefore, with the increase of $\tau^{2}$, the second probability moment and the uncertainty decrease, which improves the robustness of the system against outliers. Take the limit of JS divergence, and obtain $\tau_{h}^{2}$ according to the above equation. Finally, the Gaussian normalized product gets a new ${\left(\tau^{\prime }\right)}^{2}$.

## 4. Experiments and Discussion

### 4.1. Experimental Description

The systemic performance was evaluated by using the TUM RGB-D and EUROC MAV datasets. The system selected and improved was point-and-line-based SVO (PL-SVO) with the same depth filter as SVO, and we compare it with the state of the art: SVO/PL-SVO. All the experiments were performed on a notebook equipped with an Intel Core i5-9300H CPU (@2.40 GHz) and NVIDIA 1660Ti GPU. We ran each sequence seven times and show the average result of trajectory accuracy estimation. The evaluation indices used in the experiment were absolute trajectory error (ATE), and root means square error (RMSE) [34].

1. Root Mean Square Error (RMSE): It represents the root mean square error of the Lie algebra of the camera pose, which can describe the difference in rotation and translation between two trajectories.
2. Absolute Trajectory Error (ATE): The absolute trajectory error (ATE) is used to reflect the drift between the ground truth trajectory and estimated trajectory.
3. Root Mean Square Error of ATE: It is applied to evaluate the system accuracy. So the ATE and RMSE of the frame are defined as follows:

(23) $$F_{i}∶=Q_{i}^{-1}SP_{i}$$

(24) $$RMSE\left(F_{1:n},\Delta \right)∶={\left(\frac{1}{m}\overset{m}{\underset{i=1}{\sum }}{\left|\left|trans\left(F_{i}\right)\right|\right|}^{2}\right)}^{\frac{1}{2}}$$

- Ground-truth: $Q_{1},\ldots ,Q_{n}\in SE\left(3\right)$, subscript represents time t (or frame).
- Δ: Represents interval time; Algorithm estimates pose: $P_{1},\ldots ,P_{n}\in SE\left(3\right)$.
- S: Transformation Matrix from estimated pose to ground-truth: S$\in \mathrm{Sim}\left(3\right)$.
- $trans\left(F_{i}\right)$: Represents Translation of absolute trajectory error.
- Pose Graph: Trajectory of camera motion.
- Trans and Rot Error: The differences between translation and rotation of the monocular camera and the ground-truth are compared. Define the set of translation and rotation: $\left\{t,\delta \right\}$, where $t=\left(t_{x},t_{y},t_{z}\right)$. $\delta =\left(q_{x},q_{y},q_{z},q_{w}\right)$, where $\left(q_{x},q_{y},q_{z}\right)$ is the image, and $\left(q_{w}\right)$ is the real.
- The Number of Keyframes: A commonly method for keyframes can reduce the number of frames to be optimized and represent nearby frames. The selection criteria for keyframes are usually:
  (a) Select a keyframe at regular intervals.
  (b) Whether there are enough frames (time) from the last keyframe.
  (c) It is far enough (space) from the nearest keyframe.
  (d) Quality of tracking points (map points related).

Since there are enough valid map points in the keyframes, the selection of keyframes can also lay the foundation for the relocalization module. The accuracy of the system and the robustness of the location can also be verified by matching previous keyframes to find the camera pose.

- Initialization/Relocalization Time: Tracking and mapping in SVO are parallel threads that depend on each other. The improved SVO needs to initialize both points and lines features. Relocalization is the ability of the system from crash to recovery when the light intensity or the camera moves. Here we provide relocalization time as measurement. Both initialization and relocalization are based on valid map points, so a comparison is necessary. The code is based on C++ with the addition of a clock for timing. It is important to note that the relocalization retrieves the current camera pose by matching the previous keyframe and the projection of the map point after the tracking is lost.

### 4.2. Experimental Evaluation of TUM RGB-D Datasets

The TUM RGB-D datasets are offered by the Computer Vision Group of the Technical University of Munich and were recorded in different environments with a Microsoft Kinect RGB-D camera. Dataset Resolution: a unique motion capture system records 640 × 480, frame rate: 30 (FPS), the ground-truth is recorded by a special motion capture system.

4. Fr1_xyz uses a device that is an RGB-D camera (Microsoft Kinect RGB-D camera), and the acquired include both RGB image and depth image. The dataset is chosen because the images all become blurred when the camera moves rapidly, accompanied by repeated up-down/left-right movements, which brings some challenges to the robustness of the motion.
5. Fr2_desk uses the same equipment and records data with loop, this dataset is mainly to check the loop and the accuracy of localization.

**Figure 6:**
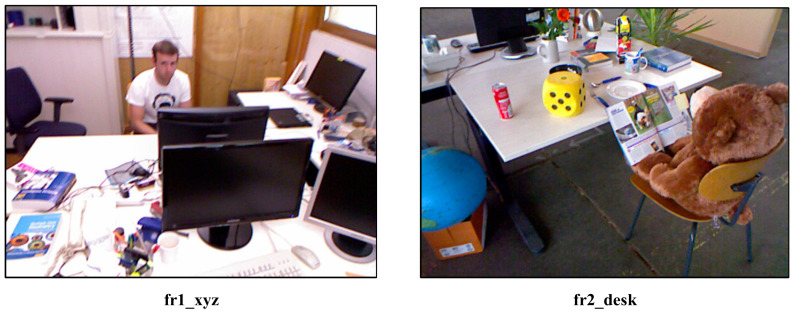
TUM RGB-D datasets.

We select two datasets for testing (fr1_xyz/fr2_desk) as shown in Figure 6.

We chose SVO, PL-SVO, and improved PL-SVO (the improved method in this article) for testing during the experiment:

SVO: Open sourced by ETH Zurich, the open-source SVO has simplified for some reason.

PL-SVO: Line features are added based on SVO, which improved accuracy and robustness in a low-texture environment.

Improved PL-SVO: Optimizing information theory (JS divergence) is added based on PL-SVO.

**Figure 7:**
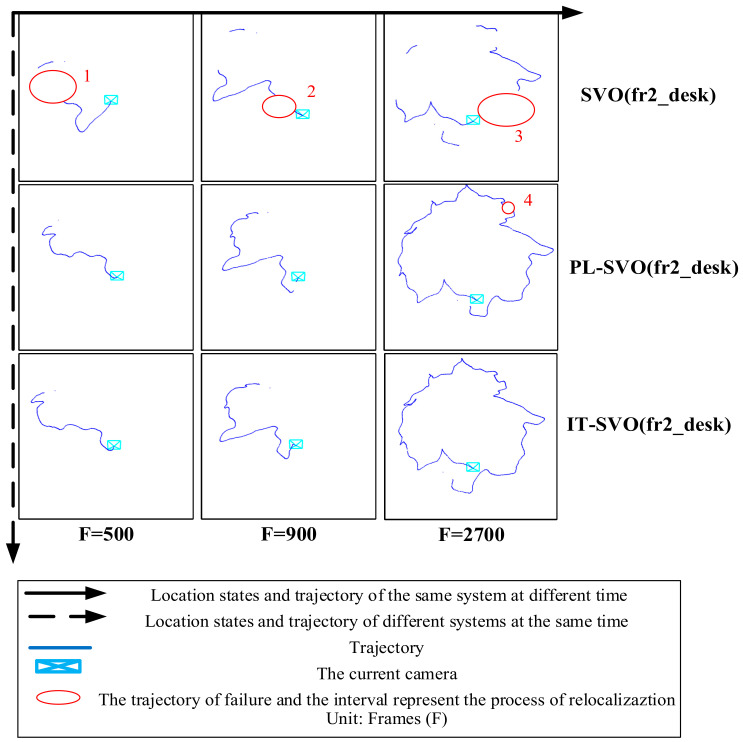
Trajectory Comparison and representation of related symbol and description (dataset: fr2_desk).

**Figure 8:**
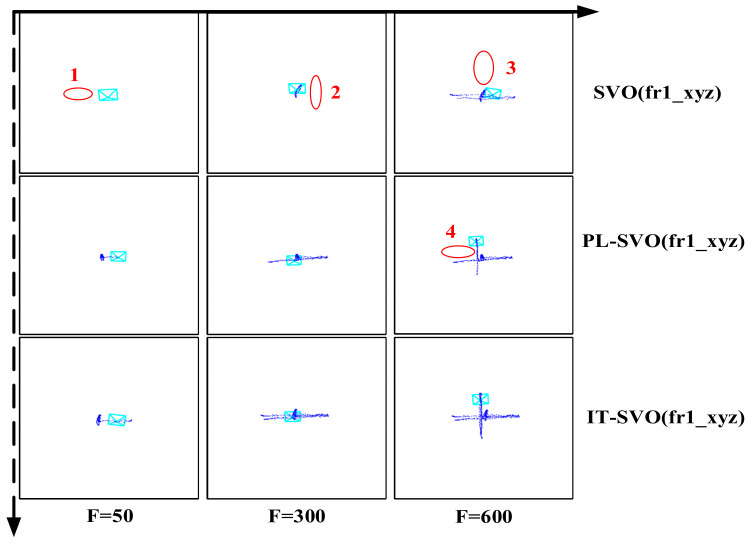
Trajectory Comparison (dataset: fr1_xyz).

The experimental results are shown in Figure 7 and Figure 8:

As shown in Figure 7 each row represents the trajectory of the same system over time/number of frame, each column represents the trajectory of different systems over the same time/number of frames.

Figure 7 shows the trajectories of three systems in the TUM RGB-D Dataset fr2_desk. The number of frames in this dataset is around 3000. The primary process is to take pictures around the desktop with a camera to verify the accuracy of the algorithm.

As seen in the first column, SVO initialization is completed successfully, but during the running process, rapid movement and motion blurring cause the SVO location to fail. As the dataset runs, the relocalization of SVO is turned on, and the current camera pose is retrieved by matching with the previous keyframes and projection of map points after the motion tracking is lost, resulting in discontinuous trajectories. PL-SVO works well but also has interruptions with large gaps at F = 900 frames. IT-SVO is superior to PL-SVO in that the overall trajectory is smoother and at F = 2500 frames the interval is smaller. Due to more keyframes are extracted, the current camera pose is restored quickly by the relocalization module.

The advantage of SVO is its speed. We tested it on airborne platform devices (DJI matrice100, NVIDIA XAVIER) and it reached 55 FPS, while on PC it reached 70 FPS. It is well known that the most feared scenes of camera motion are estimated to be pure rotation, changes in light intensity, motion blur, etc., so it is easy to lose in places where the trajectory changes sharply. Since the estimation and optimization of the poses all rely on grayscale matching, this leads to the lack of robustness of the system to illumination, while point features are selected, and the features in the scene are not sufficiently described.

In the complex environment, a large number of point features are extracted while having a simple keyframe selection strategy, resulting in unrepresentative keyframes, which can easily be terminated in the re-localization due to inadequate keyframe information.

Figure 8 shows the trajectories of three systems in the TUM RGB-D dataset fr1_xyz. Because of the partial censoring of the SVO open-source algorithm, the running results of fr1_xyz are difficult to verify. When F = 50 frames, SVO has not finished initialization, so the camera does not generate a motion trajectory. PL-SVO and IT-SVO completed the initialization at the same number of frames and successfully generated the trajectories, mainly reflected in the better relocalization and depth update module. However, with the continuation of the dataset, PL-SVO has discontinuities in the left and right corners, due to the outliers generated by the fast movement. Based on *KL* divergence, it is more challenging for SVO and KL-SVO to extract sufficient depth information from the disordered outliers. When F = 600 frames, there are also discontinuities at the up and down motion corners, while IT-SVO completes the location accurately and produce a smooth trajectory. During the algorithm, SVO has a poor localization performance for a hand-held camera (for the top view of UAV), PL-SVO can generate smooth trajectories under horizontal and vertical motion, its performance is poor at the corner, and IT-SVO completes tracking better.

We modified the SVO to improve its robustness and accuracy by adding line features to the point features. For efficiency we convert the line features to edge features, i.e., segment the line features.

First, the mapping thread provides the initial depth based on the tracking thread. Then, the build thread calculates the exact depth and feeds it back to the tracking thread to achieve the pose estimation. In this process, the JS divergence is used to optimize the mapping thread, and the mean-Gaussian filter is used to suppress the outlier when solving for map points.

We compare the performance of the improved SVO with state-of-the-art systems, i.e., SVO, PL-SVO. Table 1 shows the comparative results of RMSE for ATE. Trans, and Rot representing RMSE of relative pose error (RPE) of the translation and rotation, respectively, where “-” means tracking failure. The smallest indicate the best accuracy (the trajectory lengths of fr1_xyz and fr2_desk are 7.122 and 12.6 m, respectively).

**Figure 9:**
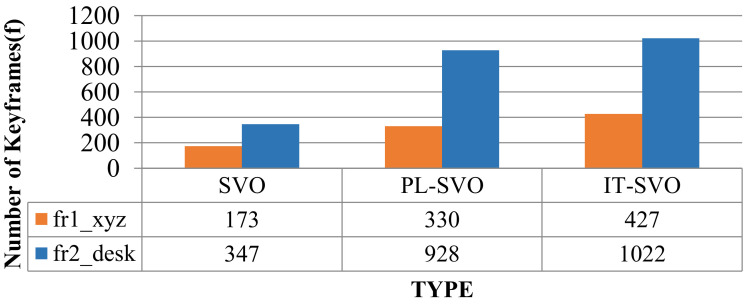
Selection of the number of keyframes (I).

As can be seen from Table 1, SVO can barely complete the location, so it is set to “-”, which represents the data is invalid-in fr1_xyz, fast steering, up-and-down movement lead to low robustness of the PL-SVO and underestimation of the scene scale. Similiarly in fr2_desk, the rapid movement of the camera results in two relocalizations. In contrast, IT-SVO has a lower RMSE of ATE and RMSE of RPE, which improves the robustness and accuracy. The number of key frames is selected by three systems as shown in Figure 9.

In Table 1. xyz/right: fr2_desk the *x*-axis represents the system type, and the *y*-axis represents the number of keyframes. In terms of the number of keyframes, it can be seen that compared with the state-of-the-art, PL-SVO, the improved SVO has a small improvement. As described in Section 4.1 Experimental Description, we know that there are many map points in the keyframes. On the contrary, the depth of the map points is obtained through the mapping thread, and both coexist with a linear relationship between the number of keyframes and the amount of information.

**Figure 10:**
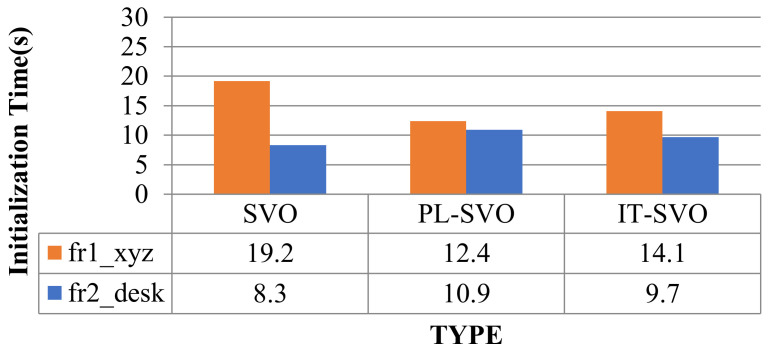
Comparison of initialization time (I).

Due to the rapid movement of the camera in fr1_xyz, SVO initialization and relocalization start almost simultaneously. PL-SVO and IT-SVO require initialization of point-and-line features, which are slower than SVO in the initialization phase. The comparison of initialization time is illustrated in Figure 10.

From the comparison of initialization time in Figure 10, it can be seen that SVO has a long initialization time for the dataset fr1_xyz and a short initialization time for fr2_desk, which needs to be explained in third aspects for this problem:

First, for fr1_xyz, we can see that the scene is single and partly white desktop and black computer. As SVO only extracts point features, although a large number of fast corners can be extracted, it is difficult to obtain map points with effective depth information, so it is necessary to perform multiple translations to achieve the initialization of the system.

Second, for fr2_desk, we can see that the scene is complex and colorful, the SVO can easily accomplish the initialization, and in contrast to PL-SVO/IT-SVO which need to extract point/line features at the same time, the initialization can be finished quickly under complex textures.

Third, PL-SVO/IT-SVO both have the same front-end (tracking thread) and have the ability of stable feature extraction. The difference in initialization time between them is mainly reflected in the back-end (mapping thread) feedback for generating valid map points. Because of the rapid movement of the camera in fr1_xyz, SVO initialization and relocalization start almost simultaneously. PL-SVO and IT-SVO require the initialization of point-and-line features. SVO runs poorly in both datasets and impossible to complete, so relocalization time is ignored and represented by “-“. The relocalization time (s) are listed in Table 2.

### 4.3. Experimental Evaluation of EuRoC Datasets

**Figure 11:**
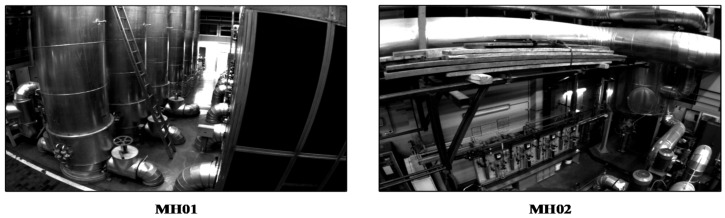
EuRoC Datasets.

We evaluate our method by using the EuRoC MAV visual-inertia datasets. The data is collected on the MAV, including a binocular camera (used to capture stereo images, FPS: 20), an IMU measurement (200 Hz) and an image resolution of 752 × 480. This experiment only uses the image sequence collected by the left camera. We select two datasets for testing (MH01/MH02). The dataset is shown in Figure 11.

(1) The content of MH01/MH02 are recorded by the UAV during flight, and the data include image from the binocular as well as the IMU. The EuRoC datasets are divided into three levels: easy/medium/high, the content of image include rotation, movement, turning, etc., and is mainly used to test the coupling algorithms of IMU and image in restricted mobile devices.
(2) The high level exists fast movements and is mainly suitable for testing algorithms of IMU+Camera, including DSO/ORB-SLAM2 are unable to complete that level currently, so we chose two datasets for easy and medium levels.

**Figure 12:**
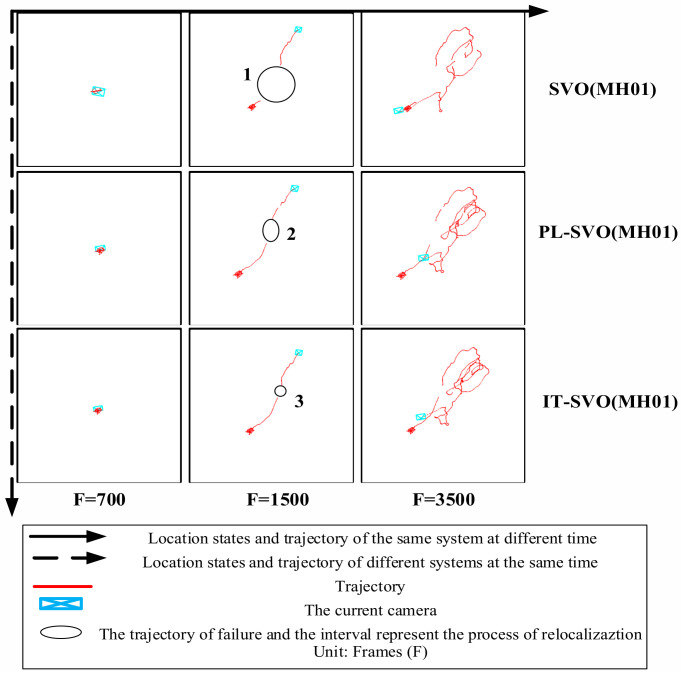
Trajectory comparison and representation of related symbol and description (dataset: MH01).

We chose the datasets collected in a factory in this experiment and chose the same SVO/PL-SVO/IT-SVO for testing. The results are shown in Figure 12.

**Figure 13:**
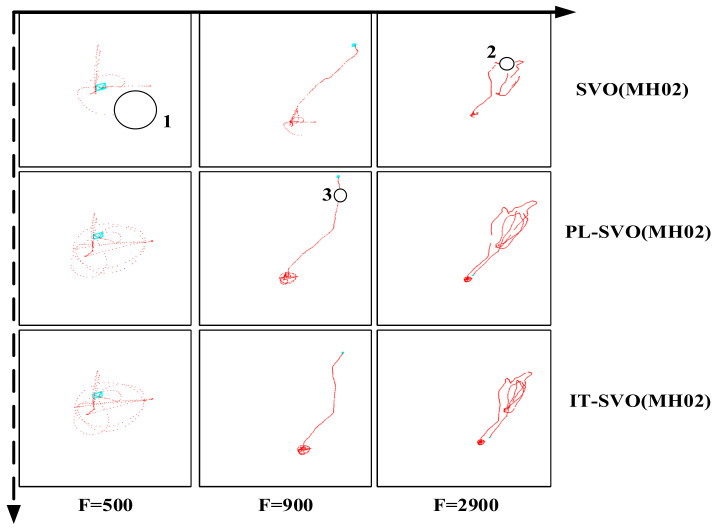
Trajectory comparison (dataset: MH02).

In Figure 12 and Figure 13, each row represents the trajectory of the same system over time/number of frames. Each column represents the trajectory of different systems over the same time/number of frames. There are three main reasons why SVO trajectory failure occur in quite smooth regions:

First, SVO is a monocular visual odometery with no closed-loop detection, which means that relocalization will not be possible when the position difference is significant.

Second, SVO compares the current frame with the previous frame to obtain a rough estimate of the pose, therefore requires the previous frame to be sufficiently accurate. If the previous frame is lost due to occlusion, blurring, etc., then the current frame will also get an incorrect result (we need a solution to effectively suppress the outlier), resulting in a poor comparison with the map, so the system will easily stop and the trajectory will have a large interruption.

Third, the smooth linear motion causes the SVO to store a considerable number of seed points, but the depth filter converges much slower than the seed point selection speed, which leads to depth mis-estimation and serious underestimation, and intuitively generates a interruption in the running.

Figure 12 shows the trajectories of three systems in the EuRoC Datasets MH01. The number of frames in this dataset is about 3700. The main content of this dataset is visual localization and navigation in a factory using IMU and camera on MAV. F = 700 is the initialization of the MAV by lifting and rotating. In the first row, due to the fast movement and insufficient point feature extraction, the initialization of SVO is low, there are more localization failures, and scale uncertainties occur during the straight-line operation. Inter-frame drift occurs when F = 3500, and the algorithm basically fails. PL-SVO and IT-SVO complete initialization, but localization fails when their running in a straight line. IT-SVO fails only on a small straight line, but it achieves tracking soon after relocalization. IT-SVO has better performance than SVO and PL-SVO in running datasets. The detailed error analysis is shown in Table 3.

**Figure 14:**
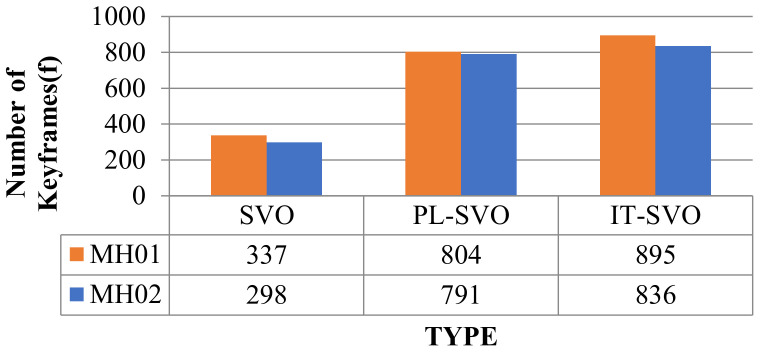
Selection of the number of keyframes (II).

In Figure 14, the SVO still has fewer keyframes, most of which are due to the instability of the system and the complex variation of the environmental characteristics. Compared with PL-SVO, the improved SVO has a little improvement. As described experimentally, the selection of keyframes brings some robustness to the system.

**Figure 15:**
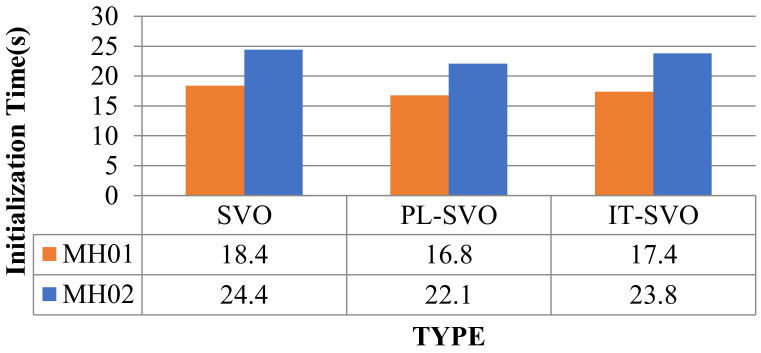
Comparison of initialization time (II).

In Figure 15, the initialization time is longer for the three systems because the initial state of the EuRoC Datasets requires initializing the camera and IMU. The initialization of the IMU requires static initialization, which has a longer initialization time than the TUM RGB-D Datasets.

The overall MH01/MH02 initialization time is higher than fr1_xyz/fr2_desk because the former requires to initialize the IMU at the same time, so the device must be stationary, while the vision-based algorithm only needs to have some translation to complete the initialization, so the initialization time is longer than the latter.

As shown in Table 4, compared with the first two systems, the improved SVO has a better relocalization function, mainly in terms of the shorter time. The relocalization time (s) are listed in Table 4.

Finally, the experiments were performed multiple times while the FPS of the processed image was used to indicate the speed. As shown in Table 5.

The processing speed on PC is higher than the onboard processor of the mobile device, but for a full-HD camera the processing speed will be slower, because in the tracking thread we only follow the third level of the pyramid, and the depth of scene will be complemented by the mapping thread.

### 4.4. Experiment and Comparison of Generalization Ability of Visual Odometry

We tested on the whole TUM RGB-D datasets, totaling twenty-three groups, and performed the corresponding experimental results and trajectory analysis. Then we choose the well-known ORB-SLAM2 for experimental comparison and select eight groups of experiments in which the proposed algorithm outperforms ORBSLAM2. Finally, the comparison video will be put to the link in the paper.

**Figure 16:**
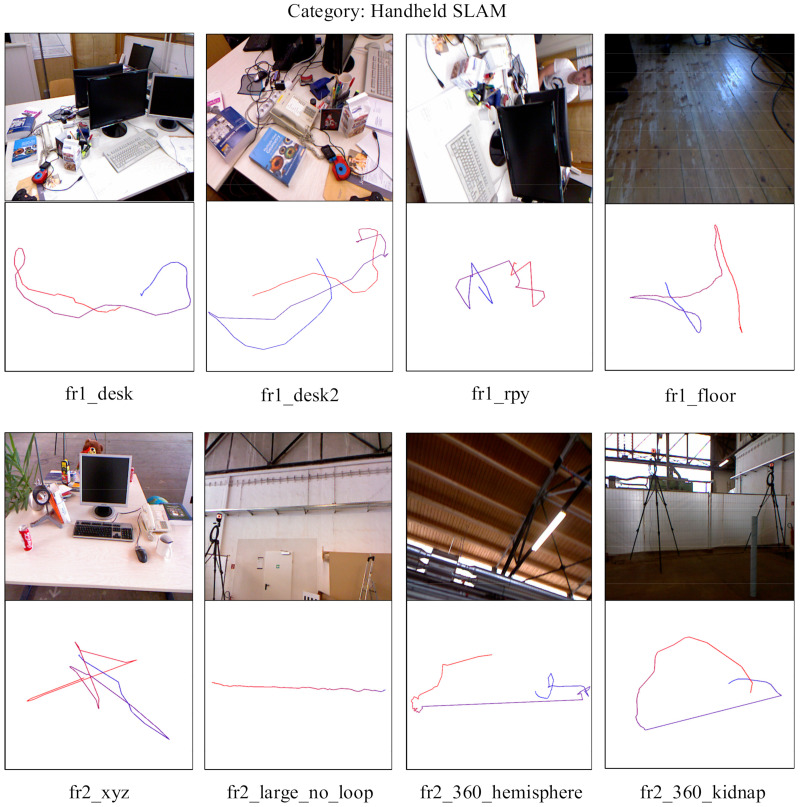
Trajectory demonstration (The top side: Raw dataset. The bottom side: Trajectory).

In the TUM RGB-D datasets, four groups of the robot SLAM category: (fr2_pioneer_360/fr2_pioneer_slam/fr2_pioneer_slam2/fr2_pioneer_slam3), all the comparison algorithms mentioned in this paper cannot be completed (including ORB-SLAM2). Meanwhile, we show the experimental screenshots of the datasets as well as the trajectories. In Figure 16, eight groups of datasets and trajectories are illustrated.

fr1/fr2: The data collected by different cameras, and some datasets that should be clarified.

fr1_rpy: This dataset represents the rotation of the camera in space to verify the rotational invariance and motion robustness of the proposed algorithm, while ORB-SLAM2 is difficult to initialize, which leads to operational failure.

fr1_floor: The main scene of this dataset is the ground (containing inhomogeneous light), where IT-SVO can achieve localization. In contrast, ORB-SLAM2 has difficulty in extracting feature points in repetitive low-texture environments and cannot complete the initialization, which leads to localization failure.

There are also several unfinished datasets containing white ground, white boxes and open field, where SVO, PL-SVO, ORB-SLAM2 and IT-SVO all fail to achieve localization (fr1_360/fr2_large_with_loop/fr3_nostructure_notexture_near_with_loop). Finally, related to that provide seven groups of demonstrations of IT-SVO outperforming ORB-SLAM2 in the Supplementary Materials, and the code will be open source in the future.

**Figure 17:**
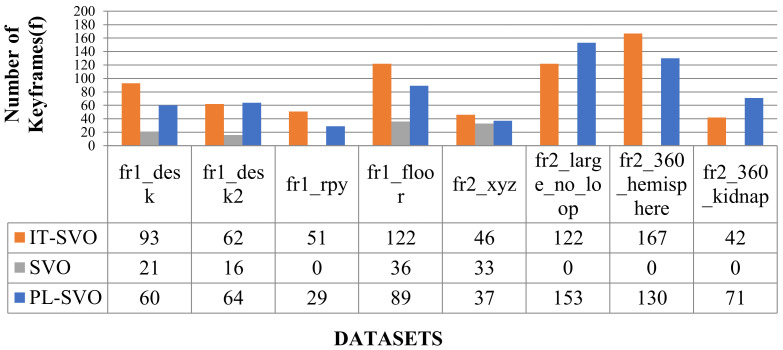
Selection of the number of keyframes (III).

**Figure 18:**
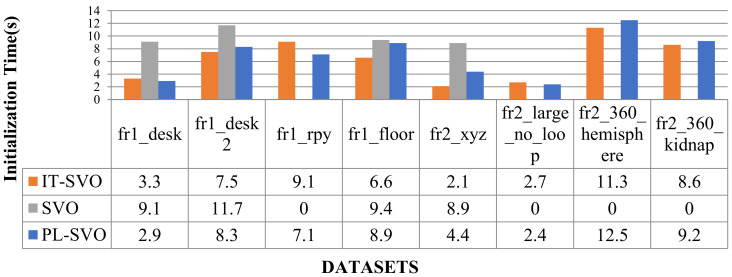
Comparison of initialization time (III).

In Figure 17 and Figure 18, we compare the number of keyframe selections and the time of initialization, as well as the detailed error analysis given in Table 6.

As shown in Figure 17, SVO does not work in the majority of environments, and in Table 6, IT-SVO outperforms PL-SVO in terms of accuracy and robustness. where a lower ATE means that the trajectory is closer to the ground-truth.

**Figure 19:**
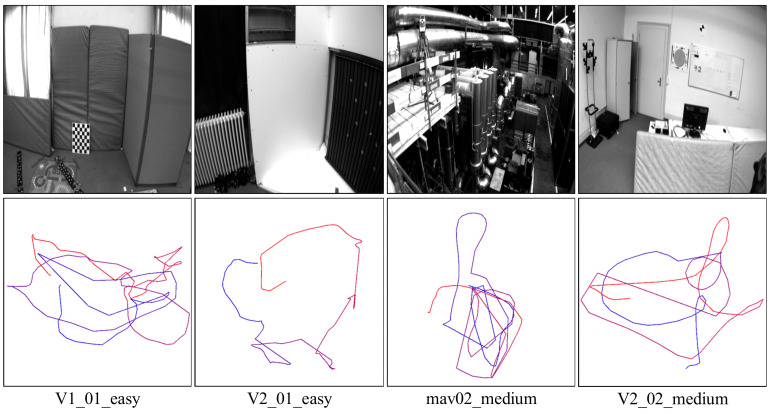
Trajectory demonstration (top: raw dataset; bottom: trajectory).

At the same time, we also tested the algorithm on the EuRoC datasets, in which due to the the difficulty level, ORB-SLAM2 that only takes the localization mode and the algorithm proposed in this paper cannot be completed, mainly because of the fast moving of the UAV. As shown in Figure 19, we tested on the additional datasets V1_01_easy, V2_01_easy, mav03_medium and V2_02_medium except for the difficulty level. The trajectory is generated from keyframes and connected under the temporal sequence. Also, it is a criterion to evaluate the accuracy of the algorithm.

**Figure 20:**
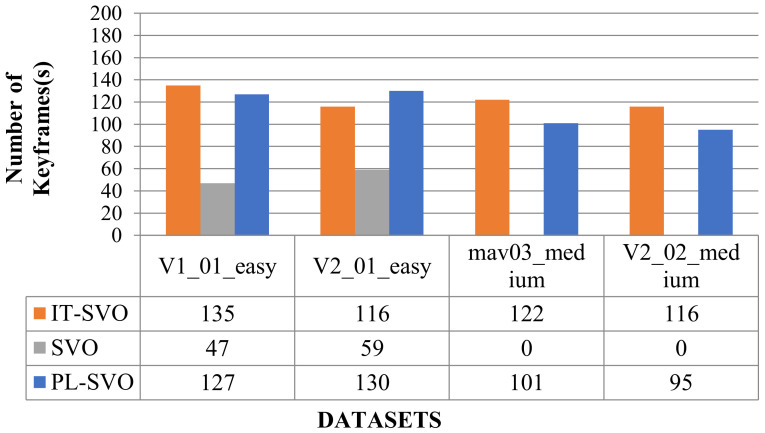
Selection of the number of keyframes (IV).

**Figure 21:**
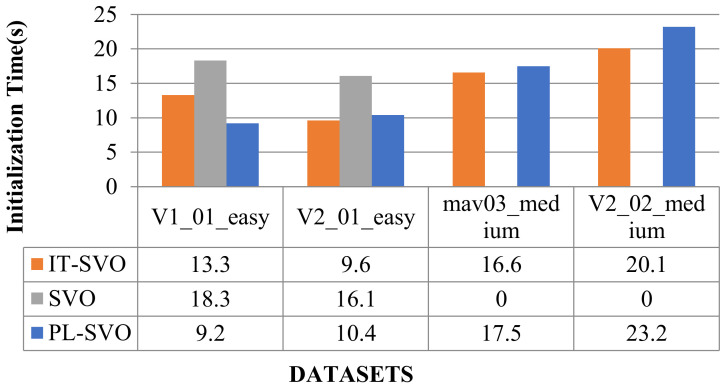
Comparison of initialization time (IV).

Figure 20 and Figure 21 show the comparison of the number of keyframes and the initialization time, respectively, followed by the corresponding error analysis in Table 7.

Finally, we performed experiments with a total of twenty-three groups and related to that seven groups of demonstrations of IT-SVO outperforming ORB-SLAM2 in the Supplementary Materials.

## 5. Results, Conclusions and Outlook

- We have optimized the depth filter module of the monocular visual odometry (SVO) by combining information theory. Through multiple sets of experimental validation experiments and error analysis, we found that the improved SVO has better localization results when combined with JS divergence in the information theory. The constructed sparse maps are more accurate and robust. Comparing the estimated trajectory with ATE, initialization time, keyframe selection, and relocalization time, it is found that the precision and robustness of the optimized SVO system are greatly improved. Therefore, our contributions are as follow:
- An efficient algorithm similar to SVO is implemented, which is also suitable for microprocessors, small devices, mobile robots, etc.
- Improved accuracy of the depth filter and sufficient information can be obtained from outliers, laying the foundation for the next update.
- Improved the system robustness and more accurate recovery of three-dimensional map points with the same computational resources.
- Increases the diversity of VO and provides a new way of methodology combined with SLAM. Simultaneously, it lays a foundation for multi-source information fusion in our future work.

We will then combine information theory to optimize and improve the VIO systems whose back-end is probability-based such as multi-state constraint Kalman filter (MSCKF). We will explore how information theory can be better played in a variable environment under combining vision and IMU to estimate the real depth of map points or camera pose. Explore and combine the applications of graph theory, probability graph models, and factor graphs in localization and navigation.

## Figures and Tables

**Table 1:** Results of SVO, PL-SVO and IT-SVO on TUM RGB-D datasets.

Sequence	SVO			PL-SVO			IT-SVO		
	Trans (m)	Rot (rad)	ATE (m)	Trans (m)	Rot (Rad)	ATE (m)	Trans (m)	Rot (Rad)	ATE (m)
fr1_xyz	-	-	-	0.2249	0.0043	1.31	0.1609	0.0029	0.84
fr2_desk				0.2214	0.0021	2.66	0.1172	0.0021	2.03

**Table 2:** Results of Re-localization time (Sequence: Two datasets. Average: The average time of re-localization between in two datasets. Note: The less time, the better capability of re-localization).

Sequence	SVO	PL-SVO	IT-SVO
fr1_xyz	-	2.776	1.082
fr2_desk		1.832	0.689
Average		2.304	0.886

**Table 3:** Results of SVO, PL-SVO and IT-SVO on EuRoC Datasets.

Sequence	SVO			PL-SVO			IT-SVO		
	Trans (m)	Rot (rad)	ATE (m)	Trans (m)	Rot (rad)	ATE (m)	Trans (m)	Rot (rad)	ATE (m)
MH01	0.8252	0.5833	9.021	0.3362	0.0191	2.39	0.3172	0.0183	1.04
MH02	1.1371	0.7270	13.24	0.5373	0.0154	3.27	0.4953	0.0167	2.03

**Table 4:** Results of re-localization on datasets.

Sequence	SVO	PL-SVO	IT-SVO
MH01	5.717	2.159	1.463
MH02	7.238	4.384	2.017
Average	6.476	3.272	1.74

**Table 5:** Algorithm operating speed (FPS) under the same standard.

	Algorithm		
	SVO	PL-SVO	IT-SVO
fr1_xyz	69	53	49
fr2_desk	76	61	65
MH01	68	50	47
MH02	61	46	51

**Table 6:** Results of SVO, PL-SVO and IT-SVO on TUM RGB-D Datasets.

Sequence	SVO			PL-SVO			IT-SVO		
	Trans (m)	Rot (rad)	ATE (m)	Trans (m)	Rot (Rad)	ATE (m)	Trans (m)	Rot (Rad)	ATE (m)
fr1_desk	0.7133	0.3826	3.47	0.1628	0.0031	1.08	0.2123	0.0036	2.01
fr1_desk2	1.4341	0.6154	4.91	0.2329	0.0047	1.37	0.2215	0.0044	1.22
fr1_rpy	-			0.2055	0.0035	0.35	0.1743	0.0032	0.31
fr1_floor	1.8755	0.7561	5.59	0.2124	0.0043	1.92	0.1917	0.0041	1.87
fr2_xyz	0.7274	0.4842	4.42	0.2179	0.0045	0.91	0.1412	0.0028	0.36
fr2_large_no_loop	-			0.1315	0.0027	1.97	0.1128	0.0020	1.85
fr2_360_hemisphere				0.1103	0.0022	1.85	0.1044	0.0019	1.79
fr2_360_kidnap				0.1227	0.0025	1.96	0.1219	0.0023	1.84

**Table 7:** Results of SVO, PL-SVO and IT-SVO on EuRoC Datasets.

Sequence	SVO			PL-SVO			IT-SVO		
	Trans (m)	Rot (Rad)	ATE (m)	Trans (m)	Rot (Rad)	ATE (m)	Trans (m)	Rot (Rad)	ATE (m)
V1_01_easy	4.3739	0.8272	13.24	0.1801	0.0092	2.66	0.1898	0.0094	2.71
V2_01_easy	4.0105	0.9194	9.02	0.1472	0.0089	1.74	0.1543	0.0091	1.79
mav03_medium	-			0.2796	0.0154	6.92	0.2533	0.0115	5.85
V2_02_medium				0.2247	0.0201	5.77	0.2134	0.0184	5.10

## Data Availability

Data is contained within the article or Supplementary Material. The data presented in this study are available in author’s github and email.

## Supplementary Materials

The demo video is implemented in the following link: https://www.mdpi.com/1424-8220/21/6/2025/s1.

## References

[1] Engel J., Sturm J., Cremers D. Camera-based navigation of a low-cost quadrocopter. Proceedings of the IEEE/RSJ International Conference on Intelligent Robots and Systems (IROS).

[2] Weiss S., Scaramuzza D., Siegwart R. (2011). Monocular-SLAM-based navigation for autonomous micro helicopters in GPS-denied environments. J. Field Robot..

[3] Mohamed S.A.S., Haghbayan M.H., Westerlund T. (2019). A Survey on Odometry for Autonomous Navigation Systems. IEEE Access.

[4] Engel J., Sturm J., Cremers J. (2014). Scale-aware navigation of a low-cost quadrocopter with a monocular camera. Robot. Auton. Syst. (RAS).

[5] Ros G., Sappa A., Ponsa D., Lopez A.M. Visual slam for driverless cars: A brief survey. Proceedings of the Intelligent Vehicles Symposium (IV) Workshops.

[6] Chen L., Hu X., Tian W., Wang H., Cao D., Wang F.Y. (2018). Parallel planning: A new motion planning framework for autonomous driving. IEEE/CAA J. Autom. Sin..

[7] Bresson G., Alsayed Z., Yu L., Glaser S. (2017). Simultaneous localization and mapping: A survey of current trends in autonomous driving. IEEE Trans. Intell. Veh..

[8] Michael B., Weiss S., Scaramuzza D. Vision based MAV navigation in unknown and unstructured environments. Proceedings of the IEEE International Conference on Robotics & Automation.

[9] Billinghurst M., Clark A., Lee G. (2015). A survey of augmented reality. Found. Trends Hum. Comput. Interact..

[10] Carmigniani J., Furht B., Anisetti M., Ceravolo P., Damiani E., Ivkovic M. (2011). Augmented reality technologies, systems and applications. Multimed. Tools Appl..

[11] Davison A.J., Reid I.D., Molton N.D. (2007). MonoSLAM: Real-time single camera SLAM. IEEE Trans. Pattern Anal. Mach. Intell..

[12] Forster C., Pizzoli M., Scaramuzza M. SVO: Fast semi direct monocular visual odometry. Proceedings of the International Conference on Robotics and Automation (ICRA).

[13] Mur-Artal R., Montiel J., Tardos J. (2015). ORB-SLAM: A versatile and accurate monocular SLAM system. Trans. Robot..

[14] Lee S.H., Civera J. (2019). Loosely-Coupled Semi-Direct Monocular SLAM. IEEE Robot. Autom. Lett..

[15] Klein G., Murray D. Parallel Tracking and Mapping for Small AR Workspaces. Proceedings of the IEEE & ACM International Symposium on Mixed & Augmented Reality.

[16] Engel J., Koltun V., Cremers D. (2016). Direct sparse odometry. arXiv.

[17] Qin T., Li P.I., Shen S.J. (2018). VINS-Mono: A Robust and Versatile Monocular Visual-Inertial State Estimator. IEEE Trans. Robot..

[18] Wang R., Wang Y., Wan W. A Point-Line Feature based Visual SLAM Method in Dynamic Indoor Scene. Proceedings of the 2018 Ubiquitous Positioning, Indoor Navigation and Location-Based Services (UPINLBS).

[19] Mur-Artal R., Tardos J.D. (2016). ORB-SLAM2: An Open Source SLAM System for Monocular, Stereo and RGB-D Cameras. arXiv.

[20] Engel J., Schps T., Cremers D. LSD-SLAM: Large-scale direct monocular SLAM. Proceedings of the European Conference on Computer Vision.

[21] Newcombe R.A., Davison A.J., Izadi S., Kohli P., Hilliges O., Shotton J., Molyneaux D., Hodges S., Kim D., Fitzgibbon A. Kinectfusion: Real-time dense surface mapping and tracking. Proceedings of the IEEE International Symposium on Mixed and Augmented Reality (ISMAR).

[22] Forster C., Zhang Z., Gassner M., Werlberger M., Scaramuzza D. (2017). SVO: Semidirect visual odometry for monocular and multicamera systems. IEEE Trans. Robot..

[23] Valiente D., Payá L., Jiménez L.M., Sebastián J.M., Reinoso Ó (2018). Visual Information Fusion through Bayesian Inference for Adaptive Probability-Oriented Feature Matching. Sensors.

[24] Huang J., Yang S., Mu T.J. (2020). ClusterVO: Clustering Moving Instances and Estimating Visual Odometry for Self and Surroundings. arXiv.

[25] Yan L.Y., Brasch N., Wang Y.D., Navab N., Tombari F. (2020). Structure-SLAM: Low-Drift Monocular SLAM in Indoor Environments. IEEE Robot. Autom. Lett..

[26] Goldberger J., Gordon S., Greenspan H. An Efficient Image Similarity Measure Based on Approximations of KL-divergence Between Two Gaussian Mixtures. Proceedings of the IEEE International Conference on Computer Vision.

[27] Zuo X.X., Xie X.J., Liu Y., Huang G.Q. (2017). Robust Visual SLAM with Point and Line Features. arXiv.

[28] Gomez-Ojeda R., Zuiga-Nol D., Moreno F.A. (2017). PL-SLAM: A Stereo SLAM System through the Combination of Points and Line Segments. arXiv.

[29] Gomez-Ojeda R., Gonzalez-Jimenez J. Robust stereo visual odometry through a probabilistic combination of points and line segments. Proceedings of the IEEE International Conference on Robotics & Automation.

[30] Pizzoli M., Forster C., Scaramuzza D. REMODE: Probabilistic, monocular dense reconstruction in real-time. Proceedings of the IEEE International Conference on Robotics and Automation.

[31] Barfoot T.D. (2017). State Estimation for Robotics.

[32] Ghaffari J.M., Valls M.J., Dissanayake G. (2018). Gaussian processes autonomous mapping and exploration for range-sensing mobile robots. Auton. Robot..

[33] Ghaffari J.M., Valls M.J., Dissanayake G. (2017). Warped Gaussian Processes Occupancy Mapping with Uncertain Inputs. IEEE Robot. Autom. Lett..

[34] Zou Y.J., Eldemiry A., Li Y.X., Chen W. (2020). Robust RGB-D SLAM Using Point and Line Features for Low Textured Scene. Sensors.

